# High-density Grid Mapping Catheter Unveiled a Deceleration Zone in a Large Ventricular Scar

**DOI:** 10.19102/icrm.2021.120112S

**Published:** 2021-01-15

**Authors:** Pedro A. Sousa

**Affiliations:** ^1^Pacing & Electrophysiology Department, Centro Hospitalar e Universitário de Coimbra, Coimbra, Portugal

**Keywords:** Deceleration zone, high-density grid, ventricular tachycardia

A 72-year-old male was referred for ventricular tachycardia (VT) ablation due to recurrent episodes of shock despite amiodarone therapy. He had a previous history of coronary artery disease with a cardiac resynchronization therapy defibrillator implanted for secondary prevention, renal insufficiency, and persistent atrial fibrillation. There was no documentation of VT on the 12-lead electrocardiogram. The transthoracic echocardiogram revealed a dilated left ventricle with an estimated ejection fraction of 36%, with an aneurism observed in the inferior wall. During the procedure, no VT was induced. Left ventricle bipolar mapping was performed with the Advisor™ HD Grid Mapping Catheter, Sensor Enabled™, while pacing from the right ventricle, and revealed a large area of low voltage (< 0.5 mV) in the inferior wall **(Figure 1A)**. Simultaneous isochronal late activation mapping was automatically performed with the AutoMap Module with Last Deflection-detection Algorithm (Ensite Precision™ Cardiac Mapping System), displayed in eight equally distributed isochrones of activation, and revealed a deceleration zone (> 3 isochrones within a 1-cm radius) inside the low-voltage area **(Figure 1B)**. The visualization of some electrograms with an amplitude as low as 0.03 mV was only possible due to the smaller electrodes with closer interelectrode spacing present in the Advisor™ HD Grid mapping catheter. The sparkle propagation map revealed a possible channel in the low-voltage area **(Video 1)** coincident with the deceleration zone. Radiofrequency was delivered in the deceleration zone and also with the goal of eliminating all local abnormal ventricular activity potentials. After ablation, a new map was created, confirming the absence of deceleration zones and local abnormal ventricular activity potentials. After one year of follow-up, the patient remained free of shocks despite the absence of antiarrhythmic drug therapy.

This case highlights the advantages of using the Advisor™ HD Grid mapping catheter for the identification of deceleration zones in areas of low voltage during ischemic VT ablation.

## Figures and Tables

**Figure 1:**
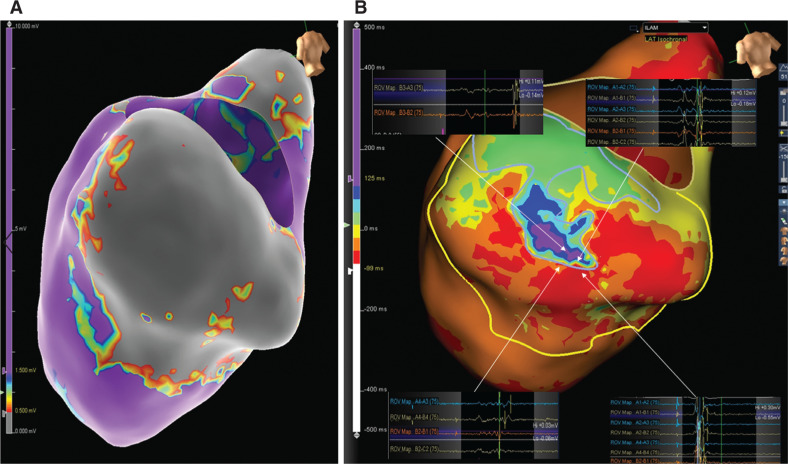
**A:** An LV bipolar map. The gray coloring indicates areas of probable scar defined as electrogram voltage below 0.5 mV and the purple coloring indicates areas of voltage above 1.5 mV. In between, yellow, green, and blue coloring represent transition areas. **B:** An isochronal late activation map, with eight equally distributed activation isochrones and with a deceleration zone as visualized by the presence of more than 3 isochrones in a 1-cm radius and, demonstrated in the electrograms. The yellow line represents the low-voltage area and the blue line indicates the late potential area.

**Video 1.** Sparkle map with the propagation wavefront revealing a channel inside the low-voltage area (yellow line) and coincident with the deceleration zone. The blue line represents the late potential area.

